# 

**Published:** 1891-09

**Authors:** 


### CONTENTS—SEPTEMBER, 1891.—VOL. VII., NO. 3.

ORIGINAL COMMUNICATIONS—

Ten Cases where Pus was Found in Abdomen: Dr. T. J. Crofford, Memphis, Tenn. .......... 78

What to do with the Insane: Dr. F. S. White, Assistant Sup't Terrell Insane Asylum .......... 85

Stab-wound in Abdomen—Laparotomy and Recovery: Dr. R. Menger, San Antonio .......... 88

Wiring the Vertebræ as a Means of Immobilization, etc.: Prof. B. E. Hadra, M. D., Galveston .......... 90

The Use of Pessaries: Dr. T. D. Wooten .......... 96

Discussion on Pessaries .......... 98

SOCIETY NOTES—

Sixteenth Quarterly Meeting Austin District Medical Society—Program .......... 100

Membership in the American Medical Association .......... 100

EDITORIAL—

Faculty of the Medical Department University of Texas. 101

Journalistic "Lagniape"—The Reading Notice Nuisance. 103

A Discrepancy in the Transactions .......... 109

MEDICAL NEWS AND MISCELLANY .......... 106

BOOK NOTICES .......... 111

PUBLISHER'S NOTES .......... 112

